# The Composition–Structure Relationship and the Formation of Fly Ash Skeletal-Dendritic Ferrospheres

**DOI:** 10.3390/molecules30071442

**Published:** 2025-03-24

**Authors:** Natalia N. Anshits, Elena V. Fomenko, Nadezhda P. Kirik, Alexander G. Anshits

**Affiliations:** Institute of Chemistry and Chemical Technology, Federal Research Center “Krasnoyarsk Science Center of the Siberian Branch of the Russian Academy of Sciences”, Akademgorodok 50/24, 660036 Krasnoyarsk, Russia; anshitsnn@mail.ru (N.N.A.); kiriknp@icct.ru (N.P.K.); anshits@icct.ru (A.G.A.)

**Keywords:** ferrospheres, ferrospinel, illite, skeletal-dendritic structure, SEM-EDS study

## Abstract

Ferrospheres (FSs) are a microspherical component of fly ash from pulverized coal combustion. The wide variations in chemical and phase composition, morphology, and the spherical design of FSs suggest their use as functional materials capable of replacing expensive synthesized materials. A general understanding of the formation of FSs from thermochemical transformations of the mineral components of the original coal is important for identifying the most promising sources of FSs with a high content of a certain morphological type active in a specific process. A systematic SEM-EDS study of the composition–structure relationship of the skeletal-dendritic FSs isolated from fly ash has revealed common routes of their formation. These FSs are formed as a result of thermochemical transformations of iron-containing minerals with the participation of aluminosilicates of the original coals. The aluminosilicate precursor that determines the skeletal-dendritic structure is illite. The crystallization of skeletal-dendritic globules occurs due to the “seed” of Al, Mg-ferrospinel formed from the thermochemical transformation of illite. The general trend of change in the structure of globules from a coarse skeletal to a fine dendritic structure is associated with a decrease in the main spinel-forming oxides content and an increase in the silicate melt viscosity.

## 1. Introduction

Magnetic complex oxides with a spinel structure form a class of compounds that are designated “spinel ferrites” owing to their structural analogy with the naturally occurring mineral MgAl_2_O_4_. These compounds correspond to the general formula MFe_2_O_4_, where M is Mn^2+^, Fe^2+^, Mg^2+^, Co^2+^, Ni^2+^, etc. Spinel ferrites are used in many electronic devices due to their unique electrical and electromagnetic properties [1,2]. They are used in radar devices as soft magnetic materials [3,4,5] and in microwave devices [6,7,8,9,10,11,12], in MRI diagnostics due to their inertness and the possibility of using them as contrast agents [13,14,15], and as catalysts and photocatalysts [16,17,18,19,20].

The main methods for obtaining oxide materials with a spinel structure are solid phase synthesis from oxides (ceramic technology) [21], coprecipitation of insoluble compounds from solutions [22], the sol–gel method [23,24] and the hydrothermal method [25,26,27]. It is important to note that each of these methods has distinct advantages and disadvantages, and their implementation is often associated with significant energy requirements and financial costs.

In recent decades, the possibility of using fly ash microspherical components, such as ferrospheres (FSs), as functional materials has been demonstrated [28,29]. The main components of FSs are ferrous phases of hematite and ferrospinel and aluminosilicate glass. Smaller amounts of martite and maghemite are also observed [30,31,32,33,34,35,36]. Narrow fractions of FSs [37] with a constant composition and reproducible magnetic properties [38] are used as catalysts for deep oxidation [39], oxidative condensation of methane [40,41,42], thermolysis of heavy oils and fuel oils [43], and as magnetic carriers for affinity sorbents for protein extraction [44].The wide variations in chemical and phase composition, morphology, and the spherical design of FSs suggest their use as durable magnetically controlled functional materials with high chemical resistance, capable of replacing expensive synthesized materials in a number of cases.

The formation of ash particles during pulverized coal combustion is the result of a number of thermochemical processes, including the fragmentation of carbon particles [45,46], mineral inclusions [47] and coalescence of internal mineral forms [48,49]. The composition, morphology of particles and their size distribution are determined by a combination of the listed processes and depend on the coal combustion conditions and the characteristics of its mineral components [50,51,52]. The formation of the globular structure of FSs occurs in the reducing environment of the carbon matrix as a result of thermochemical transformations of iron-containing and aluminosilicate mineral forms of the original coal, with the formation of droplets of high-iron melts of a complex macrocomponent composition of the FeO–SiO_2_–Al_2_O_3_–CaO–MgO system and partial crystallization of individual phases during their cooling [35,51,53]. The most frequently described are homogeneous globules of block-like, skeletal, dendritic, and plate-like structures, differing in size, the crystallite shape of the iron-containing phases, and the concentration of the glass phase [35,51,53,54]. Porous (foamy) globules with a relatively low iron content are also observed [31,37,54].

The yield of magnetic fractions from fly ash from the combustion of different coals is 0.5–18%, the iron content in them varies in the range of 20–88 wt%, and the maximum distribution of globules varies in the range of 40–150 µm [31,34,35,51,53].

Scanning electronic microscopy is one of the best and most widely used techniques for the chemical and physical characterization of fly ash [33,34,35,36,37]. SEM-EDS provides detailed imaging information on the morphology and surface texture of individual particles, as well as the elemental composition of the samples [31,55,56]. The articles provide elemental content data for individual microspheres. However, conclusions drawn from such data about the precursors of the ferrosphere phases are problematic. In order to draw sound conclusions about the precursors of the FS phases, it is necessary to analyze a larger sample of particles that are all of the same morphological type.

A general understanding of the formation of ferrospheres as a result of thermo-chemical transformations of the mineral constituents of the source coal is important to guide a targeted search for the most promising sources for the isolation of narrow fractions of ferrospheres with a high content of a specific morphological type of spheres active in a specific process. This study aimed to conduct a systematic study of the relationship between the macrocomponent composition and structure of individual skeletal-dendritic FSs isolated from fly ash from the combustion of coals from two deposits, the characteristics of their formation and the nature of the mineral precursors that determine their structure.

## 2. Results and Discussion

In order to quantitatively determine the content of different types of globules and to study the relationship between the macrocomponent composition and structure of individual skeletal-dendritic FSs, their narrow fractions isolated from fly ash from the combustion of coal from the Ekibastuz (E—0.05 mm) and Kuznetsk basins (P_2_—0.05 mm) were used.

The main components of the fractions smaller than 0.05 mm (<0.05 mm) of the E and P_2_ series were Fe_2_O_3_ (71.32 and 66.38 wt%), SiO_2_ (19.20 and 20.70 wt%) and Al_2_O_3_ (8.39 and 6.62 wt%), and the total contents of these components were 98.91 and 93.70 wt%, respectively. The CaO and MgO contents in the E series fraction (1.96 and 1.01 wt%) were lower than in the P_2_ series (2.94 wt% and 2.82 wt%). The phase composition of the E−0.05 mm and P_2_−0.05 mm series included ferrospinel (45.6 and 52.5 wt%), hematite (α-Fe_2_O_3_) (4.3 and 7.3 wt%), quartz (2.6 and 2.4 wt%), mullite (2.9 and 0.7 wt%), ε-Fe_2_O_3_ (2.8 and 2.0 wt%) and an amorphous phase (41.8 and 35.1 wt%) [57].

An analysis of SEM images of approximately 1400 globules of fraction P_2_−0.05 mm and about 900 globules of fraction E−0.05 mm showed that the content of skeletal-dendritic FSs in them was 50 and 64%, respectively. The analysis also showed that the content of block-like structures was 19% and 11%, respectively, while foamy (spongy) structures accounted for 5% and 8%, respectively. The content of plate-like globules in both fractions was <1%. The content of plerospheres in the studied fractions was 7 and 4%, respectively [58]. The presence of globules with a skeletal-dendritic structure in the composition of narrow fractions of FSs obtained from the combustion of various coals and lignite [59,60,61,62] suggests a common pathway of their formation from mineral precursors of a similar composition. To confirm this assumption, a systematic study was conducted on the relationship between the composition and structure of individual skeletal-dendritic globules obtained from the pulverized combustion of two types of Russian energy coal.

### 2.1. The Composition–Structure Relationship of Skeletal-Dendritic FSs

An analysis of the gross composition of the polished sections of the skeletal-dendritic FSs of the E series revealed that a decrease in FeO content from 87.5 to 32.5 wt% was concomitant with a monotonic increase in the content of SiO_2_ and Al_2_O_3_, ranging from 5.1 to 42.6 and 1.6 to 26.3 wt%, respectively (Table 1).

The macrocomponent composition of the P_2_ series globules changed in most of the concentration ranges. The SiO_2_ and Al_2_O_3_ contents increased in the range of 1.5–42.5 and 1.5–21.8 wt%, respectively, while the iron oxide content decreased from 87.0 to 30.5 wt% (Table 2).

The wide variation in the composition of individual FSs indicates a high degree of heterogeneity in the distribution of the mineral components from which they are formed in coal (Table 1 and Table 2). During the combustion of coal particles, the thermochemical transformation of mineral precursors in the carbon matrix leads to the formation of melt droplets. The chemical composition of these droplets corresponds to a particular type of FS.

In order to study the influence of composition on the structure of individual globules, dependences were used: [SiO_2_] = *f*[(FeO)], characterizing the iron–silicate base; and [SiO_2_] = *f*[(Al_2_O_3_)], allowing the nature of aluminosilicate precursors involved in the formation of FSs to be established, and determining their structure.

**Figure 1:**
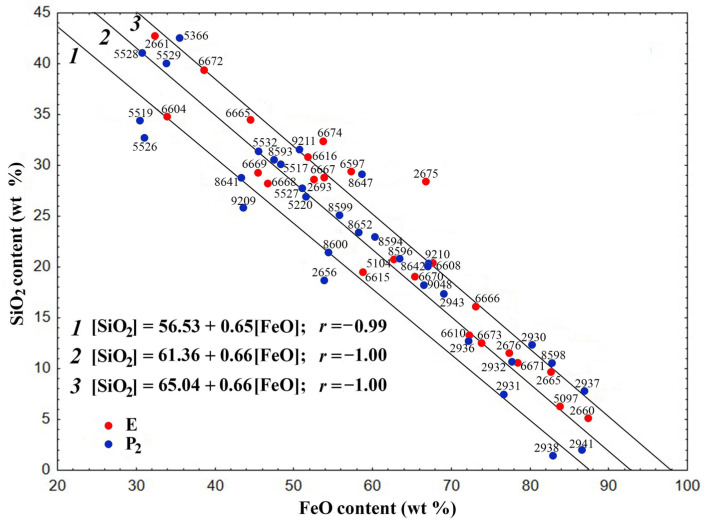
Dependence of the SiO_2_ content on the FeO content for the skeletal-dendritic FSs of series P_2_ and E.

In the dependence [SiO_2_] = *f*[(FeO)] in the gross composition of the skeletal-dendritic FSs of both series, three main groups of globules can be distinguished (Figure 1).

The composition of the globules is described by the following regression equations with correlation coefficients (*r*) of −0.99 and −1.0, respectively:

[SiO_2_] = 56.53 − 0.65[FeO] (1)

[SiO_2_] = 61.36 − 0.66[FeO] (2)

[SiO_2_] = 65.04 − 0.66[FeO] (3)

The values of the correlation coefficients indicate a strong correlation between the values of oxide contents for the regression Equations (1)–(3). The composition of the overwhelming majority of globules of both series corresponds to the second and third equations.

It must be noted that equations with similar coefficients and practically identical values of free terms describe the gross compositions of polished sections of block-like FSs formed during the combustion of coal from the Ekibastuz (series E) and Kuznetsk (series P_2_) basins [58]. These equations are as follows:

[SiO_2_] = 56.72 − 0.68[FeO]; *r* = −0.99 (4)

[SiO_2_] = 60.34 − 0.69[FeO]; *r* = −1.00 (5)

[SiO_2_] = 63.83 − 0.69[FeO]; *r* = −1.00 (6)

The decrease in FeO content and increase in SiO_2_ concentration is in accordance with the given regression equations. This suggests that iron-containing precursors do not play a decisive role in determining the structural composition of skeletal-dendritic FSs.

**Figure 2:**
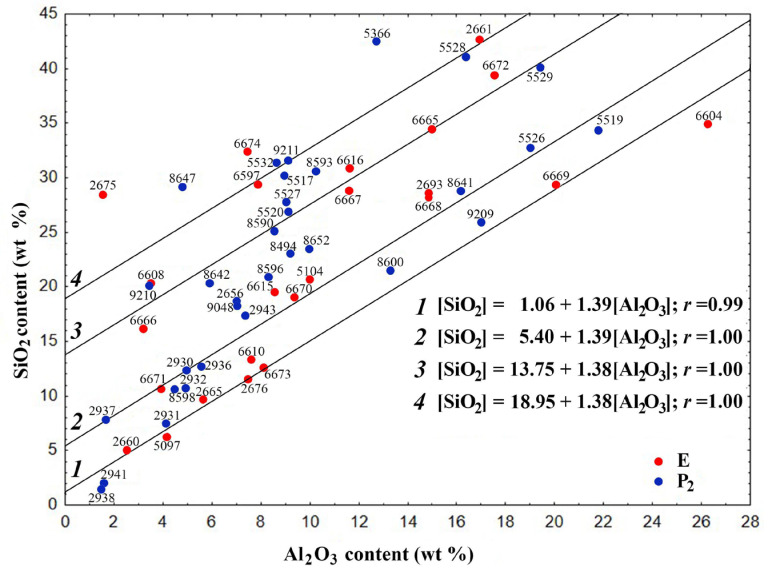
Dependence of the SiO_2_ content on the Al_2_O_3_ content for the skeletal-dendritic FSs of series P_2_ and E.

The dependence [SiO_2_] = *f*[(Al_2_O_3_)] (Figure 2) enables the estimation of the silicate modulus (SiO_2_/Al_2_O_3_) of the aluminosilicate precursor involved in the formation of FSs.

The globules can be categorized into four distinct groups, whose gross compositions are delineated by regression equations:

[SiO_2_] = 1.06 + 1.39[Al_2_O_3_] (7)

[SiO_2_] = 5.40 + 1.39[Al_2_O_3_] (8)

[SiO_2_] = 13.75 + 1.38[Al_2_O_3_] (9)

[SiO_2_] = 18.95 + 1.38[Al_2_O_3_] (10)

The correlation coefficient *r* is 0.99 and 1.0, respectively. These values suggest that there is a strong direct correlation between the values of oxide contents (the regression Equations (7)–(10)).

The coefficient in the equations indicates that the globule groups are formed with the participation of the aluminosilicate form of the mineral component of coal, with a SiO_2_/Al_2_O_3_ ratio of 1.38–1.39. The free term of the equation indicates the inclusion of an additional amount of SiO_2_ in the composition of FSs, which for the four groups is 1.06, 5.40, 13.75 and 18.76 wt%, respectively. General equations of the relationship [SiO_2_] = *f*[(Al_2_O_3_)] for the studied FSs indicate their formation along similar routes with the participation of identical aluminosilicate precursors, which can determine their structure. FSs not included in the four main groups are also apparently formed with the participation of the same aluminosilicate precursor and an intermediate or greater amount of SiO_2_ compared to the selected groups.

**Figure 3:**
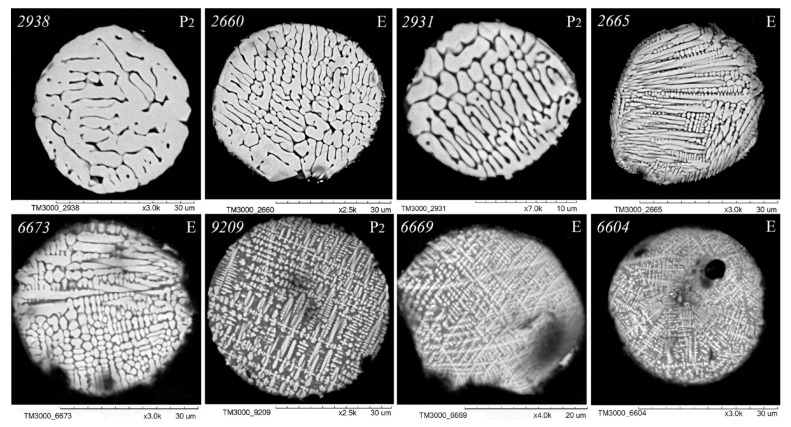
SEM images of FS polished sections of series P_2_ and E corresponding to the equation [SiO_2_] = 1.06 + 1.39[Al_2_O_3_] (Figure 2).

**Figure 4:**
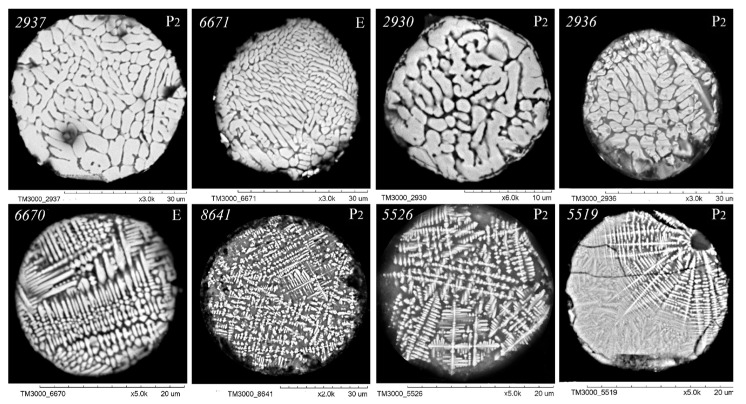
SEM images of FS polished sections of series P_2_ and E corresponding to the equation [SiO_2_] = 5.40 + 1.39[Al_2_O_3_] (Figure 2).

**Figure 5:**
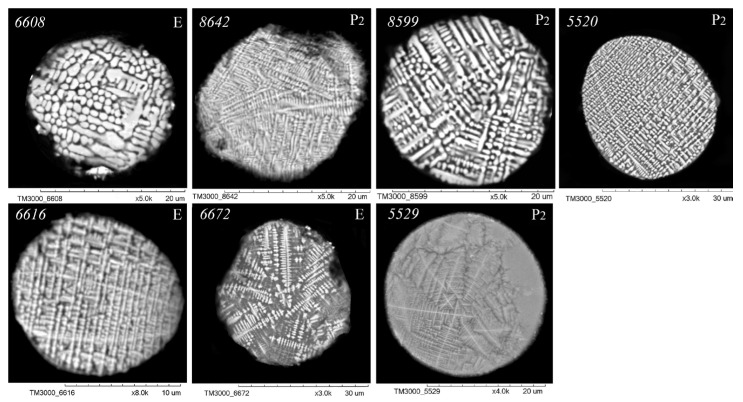
SEM images of FS polished sections of series P_2_ and E corresponding to the equation [SiO_2_] = 13.75 + 1.38[Al_2_O_3_] (Figure 2).

**Figure 6:**
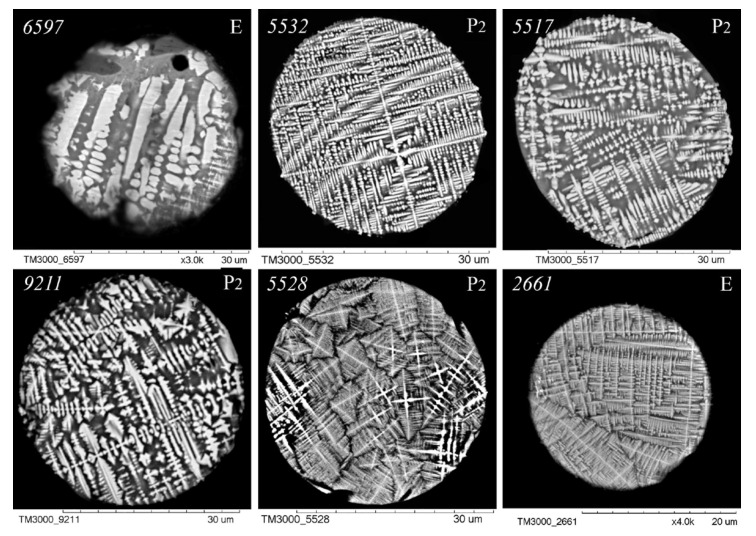
SEM images of FS polished sections of series P_2_ and E corresponding to the equation [SiO_2_] = 18.95 + 1.38[Al_2_O_3_].

As illustrated in Figure 3, Figure 4, Figure 5 and Figure 6, scanning electron microscopy (SEM) images of polished sections of globules reveal a monotonic increase in the content of SiO_2_ and Al_2_O_3_, and a decrease in the content of FeO, concomitant with changes in their structure. The globule number is indicated in the left corner of all SEM images, and in the right corner, whether they belong to the fraction of the P_2_ or E series is indicated.

An analysis of the relationship between the macrocomponent composition and the structure of polished sections of individual globules from the three aforementioned groups (Figure 3, Figure 4, Figure 5 and Figure 6) demonstrated that a decrease in FeO concentration and concomitant increase in SiO_2_ content resulted in a general tendency for the structure of the globules to change from coarse crystalline skeletal to fine crystalline skeletal-dendritic, accompanied by the simultaneous manifestation of a glass phase.

Analogous changes in the structure of the skeletal-dendritic globules with a decrease in the FeO concentration were observed for FSs formed during the combustion of coal and lignite [60]. This phenomenon can be attributed to a decline in the concentration of spinel-forming components, concomitant with an augmentation in the liquation area within the FeO–Fe_2_O_3_–SiO_2_ system melt, accompanied by an escalation in its oxidation state.

### 2.2. The Formation of Skeletal-Dendritic Structure of Globules During Thermochemical Transformations of Precursors

A comprehensive understanding of the precursors involved in the formation of FSs should be based on knowledge of the mineral composition of the original coal and the features of its thermochemical transformation. The mineral part of the coal from the Ekibastuz and Kuznetsk basins is represented by clay minerals (mainly hydromicas) 54%, quartz 28 and 14%, siderite 10 and 10.5%, calcite 5% and 2%, magnetite 2%, gypsum 2% and 7.5%, respectively [63,64]. The associations of iron-containing precursors (pyrite and siderite) with quartz, calcite or aluminosilicate minerals from different coals play an important role in the formation of FSs, which significantly facilitate the coalescence of spatially localized products of thermochemical transformation of mineral components of the original coals [65]. The presence of MgO and MnO in the globules indicates their formation from siderite containing isomorphic impurities of magnesium and manganese carbonates. The main primary product of the thermochemical transformation of siderite at 450–600 °C is wüstite (FeO) [51,66]. Subsequent melting of finely dispersed products of the decomposition of aluminosilicates, calcite and their associations with wüstite occurs at higher temperatures in the FeO–SiO_2_ and FeO–silicate–CaO systems, including low-temperature eutectics. In particular, the eutectic temperature in the FeO–CaSi_2_Al_2_O_8_–SiO_2_ system is 1070 °C, Ca_3_Si_3_O_9_—olivine 1105 °C, FeO–CaFeSiO_4_—1115 °C, and FeO–Fe_2_SiO_4_—1177 °C [35,67]. From the above, it can be concluded that the formation of FSs from siderite, pyrite and other mineral precursors occurs by thermochemical transformation in the general FeO–SiO_2_–Al_2_O_3_–CaO system with the participation of the same low-temperature eutectics. Only the primary stages of FeO formation differ; in the case of siderite, this is the decarbonization stage, and in the case of pyrite it is oxidation. This is confirmed by the general equations of the relationship between the composition of [SiO_2_] = *f*[(FeO)] and [SiO_2_] = *f*[(Al_2_O_3_)] (Figure 1 and Figure 2) and the general nature of the influence of the composition on the structure of the globules for the studied globules and FSs obtained from the combustion of coal and lignite [58,62].

**Figure 7:**
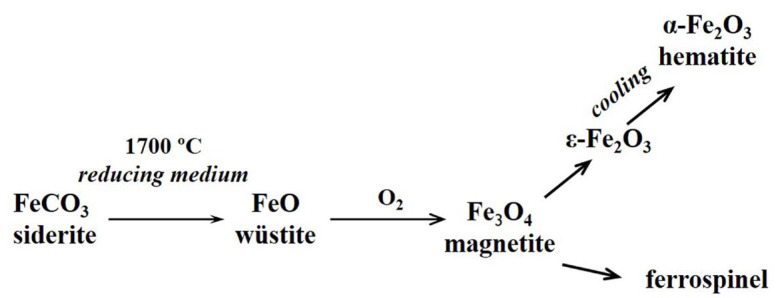
The thermochemical transformation of siderite in the carbon matrix.

Subsequent oxidation of melted droplets in areas with high oxygen content and crystallization at lower temperatures determines the phase composition of the resulting FSs. The value of melt viscosity with a decrease in the iron content from 70 to 35 wt% in the FeO—Al_2_O_3_—SiO_2_ system increases more than 20-fold at 1200 °C [68]. This complicates the crystallization of high-viscosity melts on cooling and is the reason for the high content of glass phase in FSs with a low iron content. During cooling, the melts recrystallize, which is influenced by the viscosity of the melts, the nature of the phases in equilibrium with that melt, and a change in the oxidation potential. The oxidation of Fe^2+^ to Fe^3+^ is a result of the oxidation potential change. The thermochemical transformation of siderite in the carbon matrix follows the scheme shown in Figure 7.

The aluminosilicate component of the mineral part of coal includes hydromicas and quartz. For hydromicas of the illite group with the general formula K_1−*x*+*y*_(Al, Fe^3+^)_2−*y*_(Mg, Fe^2+^)*_y_*Si_3+*x*_ Al_1−*x*_O_10_(OH)_2_, the ratio SiO_2_/Al_2_O_3_ is 1.39 [51,69], which practically coincides with the values of the coefficients in Equations (7)–(10) of the dependence SiO_2_ = *f* (Al_2_O_3_) (Figure 2). Based on this, it can be concluded that hydromicas of the illite group are involved as an aluminosilicate precursor in the formation of FS groups of skeletal-dendritic structure from ashes from the combustion of Ekibastuz and Kuznetsk coal.

As a result of their thermochemical transformation at 940–1000 °C, glass phase, quartz and iron–aluminum–magnesium spinel are formed [51,66]. The transformation mechanism of illite clay was studied by differential thermal analysis, thermogravimetric analysis and X-ray powder diffraction [70,71]. The process of thermal transformation of illite includes four main reaction processes that occur on calcination: dehydration, dehydroxylation, structural breakdown and recrystallisation. Interlayer water is driven off at 350–400 °C, followed by dehydroxylation between 450–700 °C and irreversible structural breakdown between 800–900 °C. The formation of spinel occurs at 900 °C and it continues to increase in amount and particle size with increasing temperature [70,71]. In particular, it has been shown that in the products of thermal decomposition of hydromicas of the illite group at 950 °C, the spinel content reaches 30% [51]. Obviously, the formed crystallites of Al, Mg-ferrospinel act as a “seed” during the crystallization of different compositions of melted droplets.

**Figure 8:**
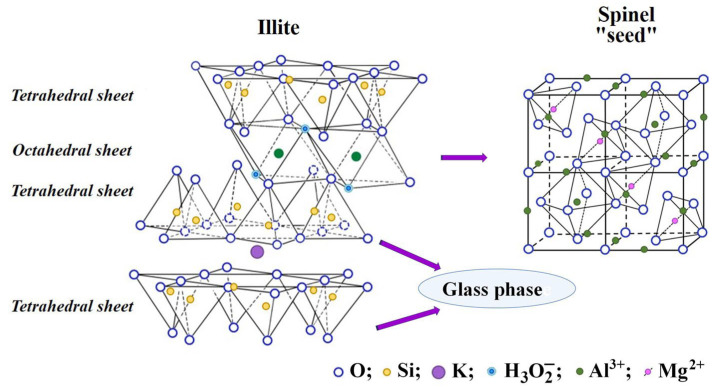
The thermochemical transformation of illite in the carbon matrix.

Illite is a crystalline mineral with a layered structure, and the basic crystalline unit of illite consists of two tetrahedral sheets and one octahedral sheet. The tetrahedral sheets consist of silicon atoms located in the cavities of the tetrahedra of oxygen atoms, while aluminum, magnesium or iron cations coordinated with hydroxyl groups are located in the cavities of the octahedra of oxygen atoms. At high temperatures, the octahedral sheets are transformed into spinel nuclei. The tetrahedral sheets are the basis of the glass phase (Figure 8).

Concurrently, the space between the crystallites is filled with iron–calcium silicate glass. In contrast to block-like FSs, all compositions of the crystallites of skeletal-dendritic globules contain fewer iron oxides and more Al_2_O_3_, SiO_2_, and MgO. The isomorphic substitution of iron by Mg^2+^ and Al^3+^ cations leads to a decrease in the parameters of the crystal lattice [72].

It is shown that the formation, growth and size of skeletal-dendritic crystals are determined by the temperature, heterogeneity and viscosity of the medium feeding the crystal growth. The viscosity of the liquid phase has a significant effect on the rate of nucleation and growth, as well as the shape of the crystals. Also, with increasing viscosity, the size of crystallites decreases. Under conditions of accelerated growth, particularly in a viscous medium, the incoming substance manages to grow only the protruding parts of the crystal (peaks and edges), mass transfer to which can be carried out at maximum speed [73,74,75,76]. This is how crystal forms arise that are classified as skeletal and dendritic. Skeletal-dendritic globules of FSs are formed as a result of transformations of iron-containing minerals with the participation of aluminosilicates, in particular illite. This process occurs as a result of an increase in the viscosity of the melt with an increase in the content of aluminum and silicon oxides and a sharp drop in temperature. Aluminum, magnesium, and calcium cations can be included in the ferrospinel lattice. The most likely substitution of Fe^2+^ for Mg^2+^ is due to the proximity of their ionic radii of 0.08 and 0.074 nm, respectively. The formation of Mg–ferrite spinel prevents its oxidation to hematite. Consequently, the “seed” in the form of Al, Mg ferrospinel crystallites is a key factor in determining the direction of crystallization of the microdroplets of the melt of the FeO–SiO_2_–Al_2_O_3_–CaO system in the form of FSs of the skeletal-dendritic type.

## 3. Materials and Methods

### 3.1. Materials

The FS narrow fractions (smaller than 0.05 mm) isolated from fly ash produced by the combustion of pulverized hard coal of grade SS (mvb) from the Ekibastuz Basin (series E) and grade T(sa) from the Kuznetsk Basin (series P_2_) were used as the study objects. The combustion process was performed in BKZ-420-140 and BKZ-320-140 boiler furnaces (flame core temperature, 1700 °C) with a dry ash removal unit at the Omsk Thermal Power Station-4. Fly ash was sampled from fields 1 and 2 of an electrostatic precipitator. The FS narrow fractions were obtained by multistage isolation from MCs involving particle size classification, followed by hydrodynamic separation to remove nonmagnetic impurities. The detailed information regarding the methods employed for the discharge fractions E—0.05 mm (Fe_2_O_3_ content: 71.32 wt%) and P_2_—0.05 mm (Fe_2_O_3_ content: 66.38 wt%) and the definitions of their chemical and phase compositions can be found in the work of [72].

For the studies of the structure and composition, separate globules were made of polished sections of FSs by way their fixation in epoxy resin with subsequent consistent polishing Struers TegraPol 15 (Struers, Ballerup, Denmark) and applying a 20 nm thick layer of platinum by the method of vacuum magnetron spraying with the EM installation ACE 600 (Leica Mikrosysteme GmbH, Vienna, Austria).

### 3.2. Characterization Techniques

The analysis of individual FS polished sections was conducted with scanning electronic microscopy (SEM) and X-ray energy dispersive spectroscopy (EDS) using a scanning electronic microscope TM-3000 (Hitachi, Tokyo, Japan) equipped with an energy dispersive X-ray spectrometer with a detector XFlash 430 H at an accelerating tension 15 kV. Mode mapping was performed using the X-ray energy dispersive microanalysis Quantax 70 (Bruker, Billerica, MA, USA). The data accumulation time exceeded 10 min, and the quality spectrum was obtained in this mode, allowing quantitative definition of the general compounds of the separate globules. The root mean square error definitions of the contents of the elements under study in the FSs were as follows in %: O 3.0–3.7, Fe 0.7–1.6, Si 0.1–0.6, Al 0.08–0.4, Ca 0.04–0.1, Mg 0.03–0.14, Na 0.03–0.07, K 0.003–0.03, Ti 0.03–0.05, and Mn 0.03–0.05. The concentrations of the elements were calculated for the content of the relevant oxides and iron for FeO, and are reduced to 100%.

## 4. Conclusions

A systematic SEM-EDS study of the composition–structure relationship in skeletal-dendritic FSs separated from two types of coal fly ash revealed common routes of their formation and the features of mineral precursor effects on their structure. The presence of groups of globules was identified, for which the gross composition of polished sections corresponds to the general equations of the relationship between the concentrations of [SiO_2_] = *f*[(FeO)] and [SiO_2_] = *f*[(Al_2_O_3_)]. It is shown that the studied FSs are formed from melted droplets of the FeO–SiO_2_–Al_2_O_3_ system during the crystallization of individual phases during their cooling. The formation of melted droplets occurs due to the successive transformation of dispersed products of thermal transformation of associates of mineral precursors such as siderite, quartz, and aluminosilicate components in the carbon matrix. In the conditions of a viscous medium with a predominance of the growth rate in one of the directions, spatially oriented skeletal, dendritic and skeletal-dendritic forms of ferrospinel crystals are formed along the peaks and edges of the crystallite. The space between crystallites is filled with the glass phase. The aluminosilicate precursor that determines the structure of the skeletal-dendritic globules is hydromica of the illite group. The crystallization of ferrospinel globules of the skeletal-dendritic type occurs due to the “seed” of Al, Mg-ferrospinel, formed as a result of the thermochemical transformation of the illite of the original coal. The general tendency of the change in the structure of FSs from the coarse crystalline skeletal type to the fine crystalline skeletal-dendritic structure is associated with a decrease in the content of the main spinel-forming oxides Fe_x_O_y_, Al_2_O_3_ and MgO in the ferrospheres’ composition.

## Figures and Tables

**Table 1:** Chemical gross composition (wt%) of skeletal-dendritic globules of the E series—0.05 mm.

Globule	SiO_2_	Al_2_O_3_	FeO	CaO	MgO	Na_2_O	K_2_O	TiO_2_	SO_3_	MnO
2660	5.05	2.51	87.47	1.36	2.26	0.10	0.05	0.12	0	0.92
5097	6.24	4.16	83.85	1.05	2.03	0.33	0.05	0.04	0	2.18
2665	9.61	5.63	82.70	0.91	0.33	0.22	0.01	0.22	0	0.25
6671	10.56	3.93	78.48	1.18	2.98	1.22	0.06	0.02	0.13	1.41
2676	11.51	7.49	77.42	1.55	0.60	0.29	0.07	0.07	0	0.78
6673	12.54	8.12	73.86	0.97	1.45	1.33	0.06	0.16	0.16	1.37
6666	16.08	3.22	73.19	0.97	3.33	1.44	0.06	0.09	0.23	1.38
6610	13.25	7.62	72.31	2.13	1.62	1.12	0.01	0.29	0.03	1.61
6608	20.33	3.54	67.67	1.20	4.28	1.23	0.03	0.04	0.14	1.53
2675	28.40	1.56	66.78	0.61	1.86	0.23	0.04	0.03	0	0.37
6670	19.02	9.38	65.45	1.11	1.71	1.13	0.04	0.24	0.21	1.68
5104	20.69	10.01	62.77	1.96	2.16	0.24	0.01	0.38	0.10	1.64
6615	19.51	8.56	58.80	4.48	4.18	1.27	0.06	1.91	0.19	0.99
6597	29.36	7.88	57.34	0.83	1.98	1.20	0.07	0.13	0.10	1.08
6667	28.77	11.62	53.87	1.13	1.69	1.11	0.07	0.11	0.22	1.39
6674	32.37	7.43	53.78	1.09	2.49	1.22	0.17	0.15	0.20	1.08
2693	28.54	14.86	52.60	0.88	1.42	0.64	0.34	0.26	0	0.38
6616	30.82	11.61	51.80	0.90	1.86	1.43	0.19	0.03	0.27	1.07
6668	28.26	14.87	46.69	4.56	2.68	0.99	0.07	0.41	0.18	1.27
6669	29.30	20.08	45.45	0.92	1.73	0.98	0.10	0.27	0.13	1.03
6665	34.44	14.97	44.51	1.44	1.27	0.89	0.07	0.81	0.21	1.36
6672	39.36	17.56	38.69	0.69	1.07	1.18	0.21	0.02	0.14	1.07
6604	34.85	26.27	33.98	1.32	0.93	1.08	0.10	0.41	0.17	0.88
2661	42.65	16.94	32.47	2.32	4.01	0.59	0.14	0.73	0.15	0.00

**Table 2:** Chemical gross composition (wt%) of skeletal-dendritic globules of the P_2_ series—0.05 mm.

Globule	SiO_2_	Al_2_O_3_	FeO	CaO	MgO	Na_2_O	K_2_O	TiO_2_	SO_3_	MnO
2937	7.79	1.68	86.97	0.86	0.74	0.56	0.11	0.00	0	1.12
2941	1.95	1.62	86.66	2.99	4.85	0.80	0.04	0.02	0.03	1.04
2938	1.48	1.47	82.93	1.87	8.71	0.73	0	0.00	0	2.75
8598	10.58	4.48	82.84	0.28	0.52	0.96	0.06	0.19	0.05	0.00
2930	12.29	4.98	80.27	0.35	0.81	0.62	0.08	0.24	0	0.35
2932	10.65	4.91	77.72	1.56	1.21	0.54	0.11	0.24	0	3.06
2931	7.42	4.14	76.66	3.37	6.19	0.82	0	0.11	0.05	1.23
2936	12.65	5.58	72.25	2.35	4.50	0.64	0.08	0.13	0	1.81
2943	17.36	7.38	69.14	1.09	3.37	0.55	0.05	0.17	0	0.85
8642	20.33	5.93	67.30	3.75	1.57	0.51	0.10	0.17	0.05	0.24
9210	20.10	3.44	67.22	2.58	2.05	1.07	0.10	0.16	0.11	3.17
9048	18.22	7.06	66.59	1.83	2.19	0.92	0.11	0.85	0	2.16
8596	20.82	8.33	63.60	2.15	2.06	1.61	0.18	0.09	0.08	1.05
8594	22.98	9.19	60.30	1.73	3.06	0.74	0.15	0.09	0.08	1.64
8647	29.07	4.79	58.75	1.75	3.86	0.65	0.23	0.03	0.02	0.82
8652	23.43	9.99	58.31	2.09	1.70	1.57	0.23	0.31	0	2.35
8599	25.07	8.56	55.83	2.74	6.32	0.69	0.35	0.40	0.03	0.00
8600	21.43	13.31	54.43	7.26	1.57	1.09	0.08	0.17	0.05	0.56
2656	18.70	7.01	53.85	6.66	11.11	0.63	0.07	0.36	0.10	1.45
5520	26.84	9.13	51.53	2.21	6.60	0.56	0.36	0.53	0.05	2.20
5527	27.79	9.07	51.18	2.24	6.36	0.36	0.32	0.27	0	2.36
9211	31.54	9.12	50.73	1.41	3.12	1.56	1.06	0.83	0.05	0.56
5517	30.07	8.95	48.35	2.53	7.51	0.31	0.09	0.16	0.03	2.01
8593	30.58	10.24	47.51	2.56	6.94	0.76	0.42	0.72	0.08	0.20
5532	31.36	8.65	45.64	2.32	7.89	0.50	0.79	1.23	0.11	1.53
9209	25.84	17.02	43.66	2.63	8.25	0.87	0.95	0.65	0.10	0.04
8641	28.77	16.17	43.38	7.99	1.94	0.58	0.29	0.20	0	0.62
5366	42.46	12.73	35.60	2.41	3.19	0.48	1.69	0.34	0	1.09
5529	40.04	19.44	33.81	1.02	3.03	0.35	1.14	0.15	0.03	0.98
5526	32.66	19.03	31.11	8.17	6.97	0.75	0.25	0.24	0.05	0.76
5528	41.07	16.37	30.84	3.34	4.52	0.56	1.83	0.65	0	0.83
5519	34.36	21.77	30.49	7.27	3.58	0.67	0.43	0.36	0.03	1.04

## Data Availability

The data presented in this study are available on request from the corresponding author.

## Abbreviations

The following abbreviations are used in this manuscript:

FSs	Ferrospheres
SEM	Scanning electronic microscopy
EDS	Energy dispersive spectroscopy
